# New biomarkers for stage determination in *Trypanosoma brucei rhodesiense* sleeping sickness patients

**DOI:** 10.1186/2001-1326-2-1

**Published:** 2013-01-07

**Authors:** Natalia Tiberti, Enock Matovu, Alexandre Hainard, John Charles Enyaru, Veerle Lejon, Xavier Robin, Natacha Turck, Dieudonné Mumba Ngoyi, Sanjeev Krishna, Sylvie Bisser, Bertrand Courtioux, Philippe Büscher, Krister Kristensson, Joseph Mathu Ndung'u, Jean-Charles Sanchez

**Affiliations:** 1 Department of Human Protein Sciences, University of Geneva, Geneva, Switzerland; 2 Department of Biotechnical and Diagnostics Sciences, College of Veterinary Medicine, Animal Resources and Biosecurity, Makerere University, Kampala, Uganda; 3 Department of Biochemistry, College of Natural Sciences, Makerere University, Kampala, Uganda; 4 Department of Biomedical Sciences, Institute of Tropical Medicine, Antwerp, Belgium; 5 Department of Parasitology, Institut National de Recherche Biomédicale, Kinshasa, D. R. Congo; 6 Division of Cellular and Molecular Medicine, St. George’s, University of London, London, Great Britain, UK; 7 INSERM UMR1094, Tropical Neuroepidemiology, Limoges, France; 8 Institute of Neuroepidemiology and Tropical Neurology, University of Limoges, School of Medicine, CNRS FR 3503 GEIST, Limoges, France; 9 Department of Neuroscience, Karolinska Institutet, Stockholm, Sweden; 10 Foundation for Innovative New Diagnostics (FIND), Geneva, Switzerland

**Keywords:** Sleeping sickness, Biomarkers, Trypanosoma brucei rhodesiense, Cerebrospinal fluid, Stage determination

## Abstract

Accurate stage determination is crucial in the choice of treatment for patients suffering from sleeping sickness, also known as human African trypanosomiasis (HAT). Current staging methods, based on the counting of white blood cells (WBC) and the detection of parasites in the cerebrospinal fluid (CSF) have limited accuracy. We hypothesized that immune mediators reliable for staging *T. b. gambiense* HAT could also be used to stratify *T. b. rhodesiense* patients, the less common form of HAT.

A population comprising 85 *T. b. rhodesiense* patients, 14 stage 1 (S1) and 71 stage 2 (S2) enrolled in Malawi and Uganda, was investigated. The CSF levels of IgM, MMP-9, CXCL13, CXCL10, ICAM-1, VCAM-1, neopterin and B2MG were measured and their staging performances evaluated using receiver operating characteristic (ROC) analyses.

IgM, MMP-9 and CXCL13 were the most accurate markers for stage determination (partial AUC 88%, 86% and 85%, respectively). The combination in panels of three molecules comprising CXCL13-CXCL10-MMP-9 or CXCL13-CXCL10-IgM significantly increased their staging ability to partial AUC 94% (*p* value < 0.01).

The present study highlighted new potential markers for stage determination of *T. b. rhodesiense* patients. Further investigations are needed to better evaluate these molecules, alone or in panels, as alternatives to WBC to make reliable choice of treatment.

## Background

Human African trypanosomiasis (HAT), commonly known as sleeping sickness, is a neglected tropical disease caused by the *Trypanosoma brucei* parasite and transmitted to humans through the bite of the tsetse fly [1]. Two morphologically identical subspecies of parasites are responsible for the disease: *Trypanosoma brucei gambiense* and *T. b. rhodesiense*[2]. In both cases, the disease progresses from a haemolymphatic first stage (S1), to a meningo-encephalitic second stage (S2). The latter reflects invasion of the central nervous system (CNS) by the parasites across the blood–brain barrier (BBB) with severe neurological complications, which can ultimately lead to coma and death, when untreated [3]. The two forms of HAT differ in their clinical presentations and geographic distribution. The *gambiense* form is widespread in Central and Western Africa and is commonly considered to be a chronic infection, which slowly progresses from the first to the second stage. The *rhodesiense* form of sleeping sickness, that affects communities in Eastern Africa, is a more aggressive illness, which rapidly progresses to the meningo-encephalitic stage [3] and accounts for less than 5% of all HAT cases [4]. Contrary to *T. b. gambiense*, for which a relatively safe drug combination has recently been introduced for treatment of S2 patients [4-6], treatment of S2 *T. b. rhodesiense* patients still relies on melarsoprol [7-9]. Melarsoprol has been reported to cause reactive encephalopathies in 8% of *T. b. rhodesiense* treated patients, which are fatal in 57% of them [8]. As a drug to safely treat both stage 1 and stage 2 patients is yet to be identified, and as S2 treatment is associated with severe side effects and toxicity [8], stage determination remains a key step in the management of patients suffering from *T. b. rhodesiense* HAT.

Staging is based on the examination of the cerebrospinal fluid (CSF) by microscopy. According to WHO, patients having ≤ 5 white blood cells (WBC) per microliter of CSF and absence of parasites are considered to be in the first stage of the disease, while patients having more than 5 WBC/μL and/or presence of parasites in the CSF are considered as S2 [10]. These methods suffer from limited specificity and reproducibility of the counting of WBC and lack of sensitivity in finding of parasites in CSF [11,12] (Dieudonné Mumba Ngoyi, personal communication).

The discovery of surrogate markers to complement or replace the counting of WBC in the staging of HAT is highly desired [11,13,14]. Many studies have focused on the staging in *T. b. gambiense* HAT [13,15-20], while less attention has been paid to *T. b. rhodesiense*, with a paucity of data on staging markers [13,21,22]. Some pro- and anti-inflammatory factors have been shown to be associated with the late stage of *T. b. rhodesiense* sleeping sickness, including IL-10, IL-6, CXCL10 and neopterin [13,21,22].

The aim of the present study was to investigate eight immune-related factors, shown to be powerful markers for stratification of *T. b. gambiense* HAT patients [13,15-20,23], as staging markers for *T. b. rhodesiense* sleeping sickness.

## Methods

### Patients

Eighty five patients (14 stage 1 and 71 stage 2) with evidence of parasites in blood, lymph or CSF were investigated in the present study (Table 1). Patients were enrolled by active or passive case finding in Malawi (NEUROTRYP study [13]) and Uganda (FINDTRYP study), in regions endemic for *T. b. rhodesiense* HAT. The studies were approved by the Ministry of Health and Population, Lilongwe, Malawi and by the Uganda National Council for Science and Technology (UNCST).

**Table 1:** Pre-treatment characteristics of the investigated patients

	**Stage 1 (n=14)**	**Stage 2 (n=71)**
**Demography**		
Sex, F (n)*	7	32
Age, years [mean ± SD]^†^	37.1 [± 19.3]	36.9 [± 15.8]
**Geographical origin**		
Malawi, n	3	27
Uganda, n	11	44
**CSF examinations**		
Trypanosome positive, n	0	64
WBC/μL (median, range)	3 [2–5]	21 [4–1140]

* Fisher’s exact test, no significant differences.

† Mann–Whitney *U* test, no significant differences.

All patients signed a written informed consent before inclusion into the study. Children (< 18 years old) or patients with altered mental status were only included in the studies after written consent of a parent or a guardian. All enrolled patients had the possibility to withdraw at any moment. Details on sample collection, inclusion and exclusion criteria of the two cohorts are reported in Additional file 1: Table 1.

CSF samples were collected by lumbar puncture and the number of WBC counted. The presence of parasites was determined using either the modified single centrifugation (Malawi) [24] or double centrifugation (Uganda) [25] methods. CSF samples were stored in liquid nitrogen at the site of collection, followed by storage at −80°C. Patients were diagnosed, staged and treated for HAT according to the guidelines of the national sleeping sickness control program of the country of sample collection.

In the present study, patients’ stage was assigned according to WHO recommendations [10], i.e., stage 1 when CSF WBC ≤ 5/μL and absence of parasite in CSF, stage 2 when CSF WBC > 5/μL and/or parasites detected in the CSF. Patients were excluded when information to classify them according to these criteria was not available.

### Immunoassays

The levels of the markers were measured in pre-treatment CSF using commercially available immunoassays (ELISA or multiplex bead suspension assay) following manufacturers’ instructions as reported elsewhere [23]. These included IgM (ICL, OR, USA), B2MG (Calbiotech, CA, USA), neopterin (BRAHMS, Germany), CXCL10 (Bio-Rad, CA, USA), VCAM-1, ICAM-1, CXCL13 and MMP-9 (R&D Systems, UK).

### Statistics

Statistical analyses were performed using IBM SPSS Statistics version 20.0.0 (IBM, NY, USA). Comparisons between groups were performed using the Mann–Whitney *U* test, setting the level of significance at 0.05. Correlations between molecules and the number of WBC were assessed through the Spearman correlation rho coefficient. ROC analyses were performed using pROC package for S+ version 8.1 (TIBCO, Software Inc.) [26]. All tests were two-tailed.

To assess the staging ability of each marker and to compare their performances at high specificity, corrected partial areas under the ROC curves (pAUC) were calculated between 90 and 100% of specificity (SP) [26]. A cut-off corresponding to 100% specificity was also computed.

To evaluate the power of the markers in predicting the presence of trypanosomes in CSF, patients were classified based on the absence (n=21) or presence (n=64) of parasites in their CSF. Complete AUC were then computed as well as the cut-off corresponding to the best combination of specificity (SP) and sensitivity (SE).

Panels of markers were obtained using an in-house software, PanelomiX, based on a method of optimization of cut-off values by iterative combination of biomarkers and thresholds (rule-induction-like method) [18]. Highly specific combinations of three molecules were computed. Those showing the highest pAUC for discrimination between stage 1 and stage 2 patients were kept. Statistical comparison between the pAUC of the panels and those of the individual markers was obtained through the Bootstrap test for two correlated ROC curves.

## Results

### Concentration of markers in patients’ CSF

The CSF levels of IgM, B2MG, MMP-9, CXCL13, CXCL10, ICAM-1, VCAM-1 and neopterin were measured on a population of 85 patients, comprising 14 stage 1 and 71 stage 2 (Table 1).

All molecules showed a higher CSF concentration in S2 patients compared to S1. IgM, MMP-9 and CXCL13 showed the highest fold increase (S2/S1 concentration ratio of 68, 12 and 187, respectively). However, the comparison using the Mann–Whitney *U* test, highlighted significant differences between the two stages for all markers (Table 2). These differences were confirmed when patients from Uganda (S1 n=11; S2 n=44) were considered separately, while only MMP-9, IgM and B2MG could significantly discriminate between Malawian S1 (n=3) and S2 (n=27) patients (Additional Figure 1). Furthermore, all markers significantly correlated (Spearman correlation) with the number of WBC counted in the CSF, the current staging method, with MMP-9, CXCL13 and CXCL10 having a rho coefficient > 0.5 (Table 2).

**Table 2:** **Concentration of markers in early (S1) and late (S2) stage *T.b. rhodesiense *patients**

**Marker**	**[S1], median**	**[S2], median**	**[S2]/[S1]**	p value*	**Spearman rho**^**†**^
**IgM [μg/mL]**	0.96	65.4	68.1	<0.0001	0.491
**MMP-9 [pg/mL]**	108.6	1309.9	12.1	<0.0001	0.554
**CXCL13 [pg/mL]**	8.2	1531.2	186.7	<0.0001	0.529
**VCAM-1 [ng/mL]**	22.6	67.3	3.0	<0.0001	0.372
**B2MG [ng/mL]**	964	3447	3.6	<0.0001	0.426
**ICAM-1 [ng/mL]**	1.99	9.6	4.8	<0.0001	0.457
**Neopterin [nmol/L]**	41.2	112.9	2.7	0.001	0.360
**CXCL10 [ng/mL]**	8.9	41.9	4.7	0.005	0.508

* Mann–Whitney *U* test.

† Correlation between the number of CSF WBC and CSF levels for each marker. Correlation was significant at 0.01 level.

**Figure 1:**
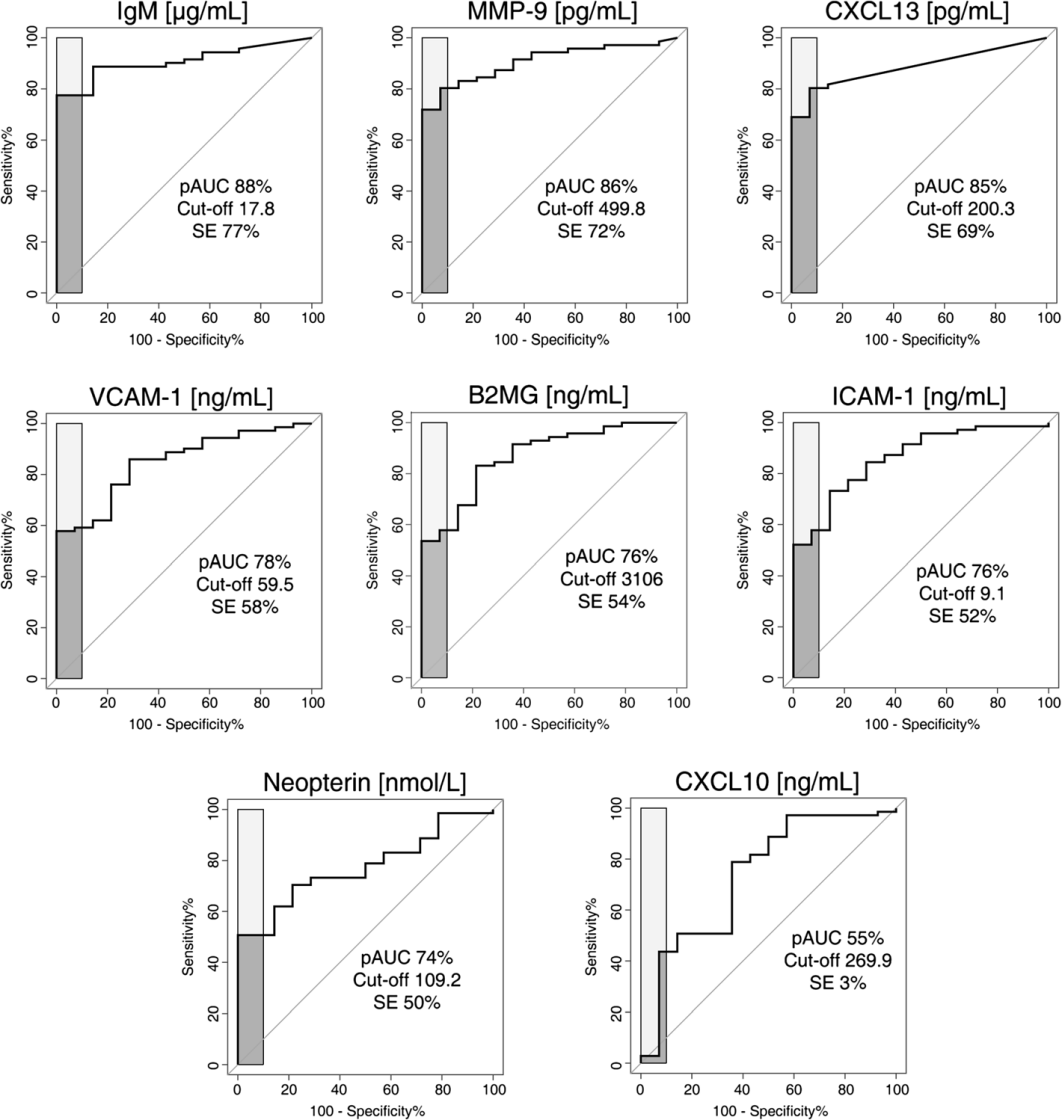
**ROC curves representing the staging abilities of the eight markers.** Dark gray areas represent the corrected pAUC between 90 and 100% of specificity obtained for each marker. Light gray zones represent a pAUC of 100%. The value of the cut-off corresponding to 100% specificity comprised within the pAUC is reported on each graph together with the corresponding sensitivity% (SE%). Additional results are reported in Additional file 1: Table 2.

### Staging ability of the markers

ROC analyses were performed to further assess the ability of the markers to discriminate between S1 and S2 patients in terms of specificity and sensitivity.

To highlight markers able to correctly rule out the highest number of S1 patients, partial AUC between 90 and 100% of specificity were calculated together with a cut-off in marker concentration at 100% of SP (Figure 1, Additional file 1: Table 2).

IgM, MMP-9 and CXCL13 had a pAUC higher than 80%, but for 100% of specificity IgM showed higher sensitivity (SE 77%, 76.6-87.3 95% CI), compared to MMP-9 (72% SE, 60.6-81-7 95% CI) and CXCL13 (69% SE, 57.8-80.3 95% CI).

A second group of markers with a pAUC between 70-80% comprised VCAM-1, B2MG, ICAM-1 and neopterin. CXCL10 was highlighted as the less accurate marker, with a pAUC of only 55% and 3% sensitivity for 100% specificity.

To further assess the ability of the eight molecules to identify patients with advanced S2 HAT, patients were classified based on the absence (T-, n=21) or the presence (T+, n=64) of parasites in the CSF. The total area under the ROC curve was considered. All markers, except neopterin and CXCL10, discriminated between T- and T+ patients with AUC > 80% and with performances comparable to those of WBC (95% CI around AUC overlapping) (Table 3).

**Table 3:** **Ability of markers to classify *T.b. rhodesiense *patients according to the presence of parasites in CSF**

**Marker**	p value*	**AUC% (95% CI)**	**Cut-off**	**SP% (95% CI)**	**SE% (95% CI)**
**IgM [μg/mL]**	< 0.0001	84.5 (73.9-95.2)	17.8	85.7 (66.7-100)	81.3 (71.9-90.6)
**MMP-9 [pg/mL]**	< 0.0001	85.2 (74.3-96)	499.8	90.5 (76.2-100)	76.6 (65.6-85.9)
**CXCL13 [pg/mL]**	< 0.0001	80.4 (69.1-91.8)	200.3	90.5 (76.2-100)	73.4 (62.5-84.4)
**VCAM-1 [ng/mL]**	< 0.0001	83.2 (73.7-92.7)	43.9	76.2 (57.1-90.5)	78.1 (67.2-87.5)
**B2MG [ng/mL]**	< 0.0001	82.0 (71.3-92.7)	1462	66.7 (47.6-85.7)	85.9 (76.6-93.8)
**ICAM-1 [ng/mL]**	< 0.0001	83.3 (73–93.6)	4.7	81.0 (61.9-95.2)	73.4 (62.5-84.4)
**Neopterin [nmol/L]**	< 0.0001	75.5 (63.9-87.1)	69.4	81.0 (61.9-95.2)	65.6 (54.7-76.6)
**CXCL10 [ng/mL]**	0.002	73.1 (59.3-86.8)	7.5	47.6 (28.6-66.7)	92.2 (84.4-98.4)
**WBC (Cells/μL)**	< 0.0001	87.4 (77.4-97.4)	6.5	71.4 (52.4-90.5)	95.3 (89.1-100)

Patients without parasites detected in CSF, n=21; patients with parasite detected in CSF, n=64.

* Mann–Whitney *U* test.

SP% = specificity%; SE% = sensitivity%; 95% CI = 95% confidence interval.

Interestingly, when the specificity and sensitivity corresponding to the best cut-off were taken into account, the three best markers, i.e. IgM, MMP-9 and CXCL13, turned out to be more specific (SP > 85%) than WBC, which in turn was more sensitive (SE > 95%) (Table 3).

### Combination of markers into panels

To evaluate whether a combination of markers could increase the staging ability, panels of three molecules corresponding to 100% specificity were calculated. Two different combinations showing the same staging performances (pAUC 94%, 89.9-97.3 95% CI; SE 87.3%, 78.9-94.4 95% CI) were obtained. Both panels comprised CXCL10 (cut-off 2.24 ng/mL) and CXCL13 (cut-off 23.3 pg/mL) in combination with either MMP-9 (cut-off 499.8 pg/mL) or IgM (cut-off 17.8 μg/mL) (Table 4). Both panels were considered positive when at least two out of three molecules were above their cut-offs.

**Table 4:** **Panels of markers for staging *T.b. rhodesiense *patients obtained through a combination of 3 molecules**

**Panel**	**Markers**	**Cut-off**	pAUC% (95% CI)*	SE% (95%CI)*	**p value**^**†**^
1	CXCL10 [ng/mL]	2.2	94 (89.9-97.3)	87.3 (78.9-94.4)	0.0001
	CXCL13 [pg/mL]	23.3			0.01
	MMP-9 [pg/mL]	499.8			0.01
2	CXCL10 [ng/mL]	2.2	94 (89.9-97.3)	87.3 (78.9-94.4)	0.0001
	CXCL13 [pg/mL]	23.3			0.01
	IgM [μg/mL]	17.8			0.02

* pAUC and SE% were calculated for 100% SP.

† Comparison between the pAUC (90-100% SP) of the panel and those of the markers individually considered obtained through the Bootstrap test for two correlated ROC curves.

Both panels are positive when ≥2 molecules are above their cut-off.

When compared to the individual molecules, the two panels were significantly more accurate for stage determination (Bootstrap test for two correlated ROC curves, p<0.05). These combinations enabled the correct classification of all S1 patients (100% SP) and 62 out of 71 S2 patients (87% SE).

## Discussion

Stage determination in *T. b. rhodesiense* sleeping sickness patients is a critical step in ensuring that the appropriate treatment is used [14]. An imperfect gold standard for staging and the lack of a safe S2 drug highlight the need for new tools for staging this form of disease [27,28]. In the present study we investigated, on a small population of patients suffering from *T. b. rhodesiense* HAT, a number of molecules (MMP-9, CXCL10, CXCL13, IgM, neopterin, ICAM-1, VCAM-1 and B2MG) known to be over-expressed in the CSF of late stage *T. b. gambiense* patients [23]. Since melarsoprol is still the only treatment for S2 *rhodesiense* patients, we evaluated their staging ability as highly specific markers, to try to limit unnecessary exposure of patients to this toxic drug. IgM, MMP-9 and CXCL13 were shown to be the most accurate discriminators between early and late stage disease (pAUC ≥ 85%) and showed the same accuracy as WBC in distinguishing between patients having parasites in their CSF from those without. Furthermore, combination of the molecules into panels of three markers (IgM-CXCL13-CXCL10 or MMP-9-CXCL13-CXCL10) significantly increased the staging accuracy, leading to the correct classification of all S1 patients and 62 out of 71 S2 patients.

All the markers investigated here are known to be involved in the immune response elicited by the presence of the parasite in the host. Interestingly, a different behavior of the 8 molecules was observed in *T. b. gambiense* patients, which may reflect the differences in immunopathogenesis [29] and clinical presentation [3,30] of the two forms of HAT. It has already been proposed, for example, that different activation pathways of macrophages and astrocytes may take place in the two forms of HAT [22]. Such differences may be responsible of the less accurate staging ability of neopterin on *T. b. rhodesiense* patients, compared to its very high staging power on *T. b. gambiense* patients.

The role of IgM, the best individual marker in the present study, in disease progression has been extensively studied. An increased CSF concentration of IgM of intrathecal origin was shown to be a good indicator of brain involvement in HAT [15], leading to the development of a rapid latex agglutination test (Latex/IgM) for stage determination in the field [31]. However, when assessed under field conditions, this assay did not represent an advantage compared to counting of WBC [31]. Furthermore, when used for evaluation of the outcome after treatment, IgM levels were not an optimal indicator of recovery due to their slow normalization [32]. Studies in animal models have shown that HAT meningo-encephalitis is characterized by an increased number of leukocytes in the CNS [33]. CXCL13, also known as BCA-1, is a chemokine mainly produced by dendritic cells [34], which specifically attracts B and T lymphocytes to the site of inflammation [35]. Its over-expression in CSF has been associated with increased WBC and intrathecal production of immunoglobulins in many pathological conditions [36,37], including late stage *T. b. gambiense* HAT [19]. On the other hand, MMP-9 (matrix-metalloproteinase 9), an enzyme involved in tissue homeostasis and remodeling [38,39], has been extensively studied in a number of pathologies affecting the CNS [39-42], in addition to *T. b. gambiense* HAT [17]. Due to its ability to degrade β-dystroglycan, this protein has been proposed to be involved in the passage of leukocytes through the *glia limitans* to reach the brain parenchyma [43]. However, the temporal relationship between the events leading to CNS invasion and the appearance of various signs and symptoms of nervous system dysfunction needs to be investigated further.

The markers investigated in the present study were combined into panels in order to increase their accuracy in stage determination. The utility of this approach to achieve a better diagnostic accuracy has already been shown [18,44]. Using this method, we highlighted highly specific combinations comprising CXCL13, CXCL10 and MMP-9 or IgM. Interestingly, CXCL10 was present in both panels. This molecule was not efficient in staging *T. b. rhodesiense* patients when considered individually. However, when combined to CXCL13 and MMP-9 or IgM, it helped in reaching a significantly increased staging accuracy. This chemokine, which specifically attracts T lymphocytes to the site of inflammation [45], was reported to be produced by activated astrocytes in trypanosome-infected mice [46]. The activation of astrocytes and macrophages are early events in stage 2 infection [47-49], suggesting that CXCL10 may represent an early indicator of CNS involvement in HAT.

Interestingly, the markers did not show the same staging performances when assessed on patients classified according to their geographic origin (i.e. Malawi or Uganda). Although the low number of Malawian S1 patients (n=3) certainly represents a bias and may be responsible for the differences observed, this result could reflect the variable clinical presentation of *rhodesiense* disease observed in different foci [27]. This may suggest that potentially different markers will be needed to stage *T. b. rhodesiense* patients according to their geographical origin and the parasite strain.

The present study has a number of limitations that should be considered. First, the data presented resulted from analyses on a small number of patients. This is a common problem associated to the investigation of this form of HAT. Collecting samples from *T. b. rhodesiense* patients is considerably difficult, not only due to the lower incidence of this disease compared to the *gambiense* form, but also as a consequence of a less effective active screening, since the CATT test can only detect *T. b. gambiense* cases [50]. To further evaluate the staging properties of the markers, larger cohorts of patients should be investigated. Moreover, due to the reported differences between *T. b. rhodesiense* HAT among foci, the results presented here should be validated in a more controlled set of patients (i.e. in which the same parasitological examinations were performed).

Another drawback could be represented by the choice of selecting highly specific markers, with the consequence of compromising the sensitivity. Management of *T. b. rhodesiense* patients is far from being optimal, thus both choices of high specificity or sensitivity would be associated either to a risk of missing the diagnosis of late stage patients, or to the exposure of S1 patients to a highly toxic stage 2 drug, respectively. However, it should be emphasized that a new staging biomarker for *rhodesiense* HAT would be combined with the detection of parasites in CSF, which would increase the sensitivity, and with clinical evaluation of the neurological status of the patients.

The absence of information on neurological signs exhibited by patients in the present study prevented an efficient assessment of the association between the levels of the markers and the signs of CNS involvement. This aspect is particularly important in the light of a recent publication on *T. b. rhodesiense* HAT reporting the poor association between disease progression, the levels of a number of cytokines and patients’ neurological manifestations [51].

The 8 markers investigated here behaved differently when assessed on *T. b. gambiense* or *T. b. rhodesiense* samples, underlining the differences between the two forms of disease, and suggesting that potentially new *rhodesiense*-specific markers could be discovered.

Despite the high staging accuracy shown by the combinations of markers described in the present study (i.e. CXCL10-CXCL13-MMP-9 and CXCL10-CXCL13-IgM), their translation into a rapid field diagnostic test could be difficult, due to a potential increase in the costs of production, suggesting that deeper investigations should be performed. The individual staging power of the molecules should be assessed on a larger cohort of *T. b. rhodesiense* HAT patients, including CSF samples collected during the post-therapeutic follow-up, and the possibility of their translation into a point-of-care test for stage determination in the field should be evaluated. Furthermore, their study in animal models, as already done for IL-10 [52], could help in the further characterization of the role of these markers in disease progression.

## Conclusions

The results presented in this work on *T. b. rhodesiense* sleeping sickness highlight the potential utility of IgM, MMP-9 and CXCL13, alone or combined with CXCL10 in staging patients. We believe that this work has paved the way for further investigations on the role of these markers in detecting the meningo-encephalitic stage of *T. b. rhodesiense* HAT, and therefore making a more accurate choice of treatment.

## Abbreviations

HAT: Human African trypanosomiasis; WBC: White blood cells; CSF: Cerebrospinal fluid; ROC: Receiver operating characteristic; AUC: Area under the ROC curve; pAUC: Partial AUC; S1: Stage 1; S2: Stage2; CNS: Central nervous system; WHO: World health organisation; NECT: Nifurtimox-eflornithine combination therapy; SP: Specificity; SE: Sensitivity; CI: Confidence interval.

## Competing interests

Joseph Ndung’u is an employee of the Foundation for Innovative New Diagnostics (FIND). Veerle Lejon, Philippe Büscher and Sanjeev Krishna were consultants for FIND at the time of the study. All other authors declare that they have no competing interests.

## Authors’ contributions

NTi, AH, JCS, VL, JMN conceived and designed the experiments. NTi and AH performed the experiments. NTi, AH, JCS, NTu and XR analyzed the data. EM, JCE, DMN, PB, VL and KK collected samples. VL, SK, JMN, PB, KK helped in data interpretation. All authors have either participated in writing or reviewing of the manuscript.

## Supplementary Material

Additional file 1 **Table 1.** Details of the studies from which samples were obtained. **Table**2**.** Detailed calculation for the evaluation of the staging ability of the eight markers. **Figure**1**.** Comparison of the levels of the markers between stage 1 (S1) and stage 2 (S2) *T.b. rhodesiense* patients classified according to the country of sample collection. For each country, differences between S1 and S2 were assessed using the Mann-Whiney *U* test. * corresponds to a p value < 0.05; ** corresponds to a p value < 0.001; *** corresponds to a p value < 0.0001. Click here for file
