# Supplementary material for: New biomarkers for stage determination in Trypanosoma brucei rhodesiense sleeping sickness patients
Source: Clin Transl Med. 2013 Jan 7;2:1. doi: 10.1186/2001-1326-2-1 (PMC3561069; doi:10.1186/2001-1326-2-1)
Supplement: Additional file 1 — Table 1. Details of the studies from which samples were obtained. Table2. Detailed calculation for the evaluation of the staging ability of the eight markers. Figure1. Comparison of the levels of the markers between stage 1 (S1) and stage 2 (S2) T.b. rhodesiense patients classified according to the country of sample collection. For each country, differences between S1 and S2 were assessed using the Mann-Whiney U test. * corresponds to a p value < 0.05; ** corresponds to a p value < 0.001; *** corresponds to a p value < 0.0001. [file 2001-1326-2-1-S1.pdf]

**Additional Table 1** Details of the studies from which samples were obtained

| | **Malawi (NEUROTRYP study, *Amin et al. 2010*)** | **Uganda (FINDTRYP study)** |
|---|---|---|
| **n S2 / n total** | 27/30 | 44/55 |
| **Site of sample collection** | Rumphi region | Lwala and Serere Health Centres |
| **Inclusion criteria** | - Detection of trypanosomes in any body fluid<br>- Age ≥ 12 years old<br>- Written informed consent | - Detection of trypanosomes in any body fluid<br>- Written informed consent |
| **Exclusion criteria** | - Moribund conditions<br>- Haemorrhagic CSF<br>- Low probability of patient coming back for follow-up | - No major exclusion criteria since the study did not implicate invasive procedures other than those applied for HAT diagnosis in clinical practice |
| **Method for trypanosome detection in CSF** | Modified single centrifugation (*Miezan et al. 2002*) | Double centrifugation (*Cattand et al. 1988*) |
| **Treatment of S1 patients** | Suramin | Suramin |
| **Treatment of S2 patients** | Melarsoprol 10 days | Melarsoprol 10 days |

S1 = S1 patients; S2 = stage 2 patients

**Additional Table 2** Detailed calculation for the evaluation of the staging ability of the eight markers

| Marker | pAUC (95% CI) | Cut-off | SP% | SE% (95% CI) |
|---|---|---|---|---|
| **IgM [µg/mL]** | 88.1 (83.7-95.6) | 17.8 | 100 | 77.5 (67.6-87.3) |
| **MMP-9 [pg/mL]** | 86.4 (81.0-94.1) | 499.8 | 100 | 71.8 (60.6-81.7) |
| **CXCL13 [pg/mL]** | 85.4 (79.2-93.7) | 200.3 | 100 | 69.0 (57.8-80.3) |
| **VCAM-1 [ng/mL]** | 78.0 (72.3-87.5) | 59.5 | 100 | 57.8 (46.5-69) |
| **B2MG [ng/mL]** | 76.2 (70.4-90.4) | 3106 | 100 | 53.5 (42.3-64.8) |
| **ICAM-1 [ng/mL]** | 75.6 (70.4-89.9) | 9.1 | 100 | 52.1 (40.9-63.4) |
| **Neopterin [nmol/L]** | 74.1 (68.8-84.4) | 109.2 | 100 | 50.7 (39.4-62) |
| **CXCL10 [ng/mL]** | 55.0 (47.4-77.8) | 269.9 | 100 | 2.8 (0-7.0) |

Partial AUC (pAUC) were calculated between 90 and 100% of specificity
SP% = specificity%; SE = sensitivity%; 95% CI = 95% confidence interval

**Additional Figure 1**

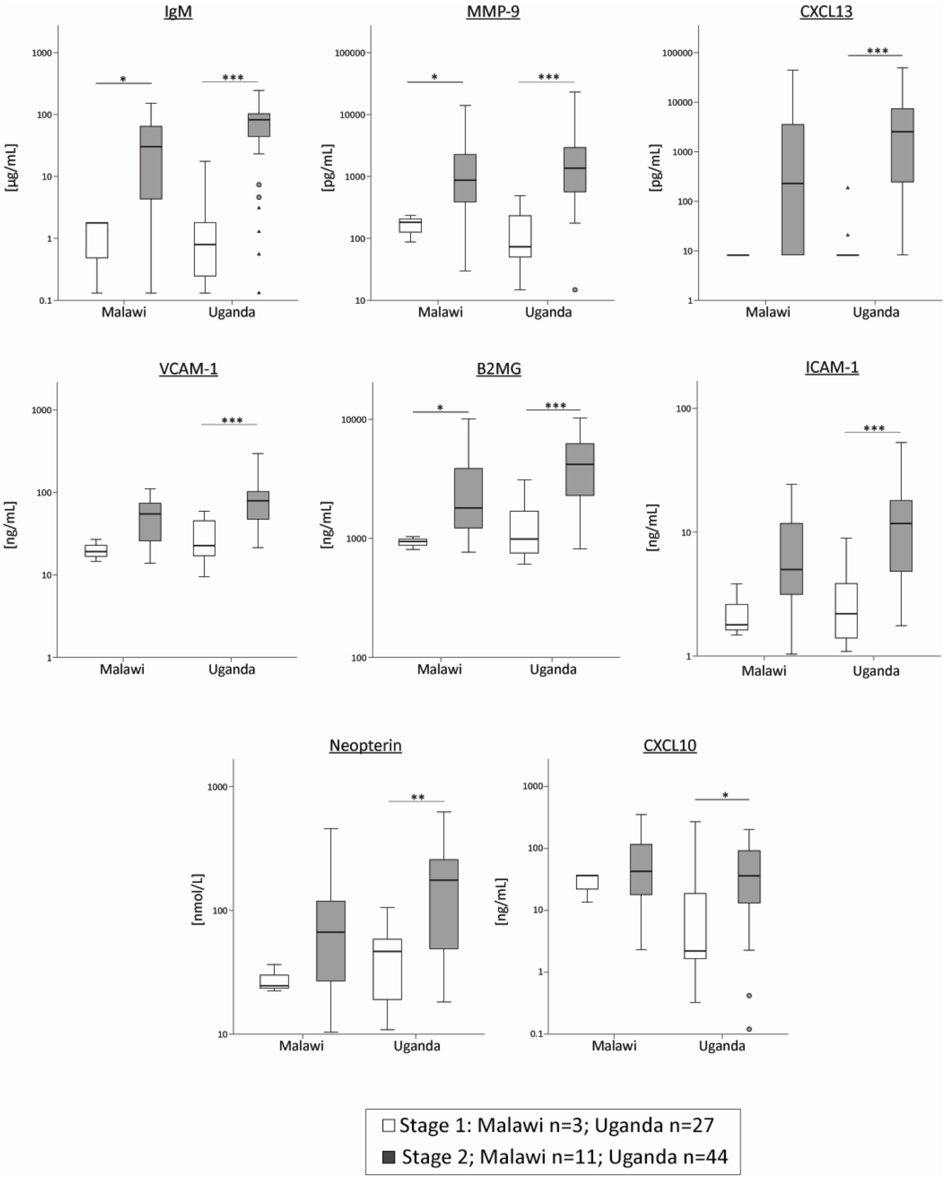


Comparison of the levels of the markers between stage 1 (S1) and stage 2 (S2) patients classified according to the country of sample collection.

For each country, differences between S1 and S2 were assessed using the Mann-Whiney *U* test.

* corresponds to a p value < 0.05; ** corresponds to a p value < 0.001; *** corresponds to a p value < 0.0001
